# The Use of Signal-Transduction and Metabolic Pathways to Predict Human Disease Targets from Electric and Magnetic Fields Using *in vitro* Data in Human Cell Lines

**DOI:** 10.3389/fpubh.2016.00193

**Published:** 2016-09-07

**Authors:** Fred Parham, Christopher J. Portier, Xiaoqing Chang, Meike Mevissen

**Affiliations:** ^1^National Institute of Environmental Health Sciences, Research Triangle Park, Durham, NC, USA; ^2^Environmental Health Research, Thun, Switzerland; ^3^Division of Veterinary Pharmacology and Toxicology, Vetsuisse Faculty, University of Bern, Bern, Switzerland

**Keywords:** electromagnetic fields, genomics, microarray gene expression, gene set analysis, signal-transduction pathways, human disease, metabolic pathways

## Abstract

Using *in vitro* data in human cell lines, several research groups have investigated changes in gene expression in cellular systems following exposure to extremely low frequency (ELF) and radiofrequency (RF) electromagnetic fields (EMF). For ELF EMF, we obtained five studies with complete microarray data and three studies with only lists of significantly altered genes. Likewise, for RF EMF, we obtained 13 complete microarray datasets and 5 limited datasets. Plausible linkages between exposure to ELF and RF EMF and human diseases were identified using a three-step process: (a) linking genes associated with classes of human diseases to molecular pathways, (b) linking pathways to ELF and RF EMF microarray data, and (c) identifying associations between human disease and EMF exposures where the pathways are significantly similar. A total of 60 pathways were associated with human diseases, mostly focused on basic cellular functions like JAK–STAT signaling or metabolic functions like xenobiotic metabolism by cytochrome P450 enzymes. ELF EMF datasets were sporadically linked to human diseases, but no clear pattern emerged. Individual datasets showed some linkage to cancer, chemical dependency, metabolic disorders, and neurological disorders. RF EMF datasets were not strongly linked to any disorders but strongly linked to changes in several pathways. Based on these analyses, the most promising area for further research would be to focus on EMF and neurological function and disorders.

## Introduction

The worldwide use of mobile phones has aroused concern about possible health effects of radiofrequency electromagnetic fields (100 kHz–300 GHz; RF EMF) (1, 2). Even though extensive research on possible effects of extremely low-frequency electromagnetic fields (ELF EMF) has been done, the underlying mechanism(s) still remain unknown (3, 4).

Many studies have been performed on identifying genes being involved in biological effects caused by ELF and RF EMF using a variety of mammalian cell lines and primary cells. Most of these studies were hypothesis-driven and demonstrated changes in the expression of a limited number of genes, especially those involved in stress response (5–18), and cell cycle regulation and apoptosis (19–23), suggesting an upregulation or downregulation of the genes involved. A few studies tried to pinpoint signal-transduction pathways involved in stress response. These investigations gave evidence that RF EMF activate the mitogen-activated protein kinase (MAPK) stress response pathway (5, 24, 25). The extracellular signal-regulated kinases (ERK) 1 and 2 are MAPKs that are also important in cellular proliferation, differentiation, and survival.

The importance of cellular context was demonstrated through comparison of the effects of EMF in different cell lines (10).

In contrast to the approach investigating EMF on a limited number of genes, studies evaluating changes in transcription profiling have been performed to speed up the identification of genes responding to EMF. Several transcriptomics studies have been performed, and the outcome has been reviewed (26). New methods for analyzing high throughput studies can be used to extract additional insight from these data. The use of analytical tools/databases allow for integrated analyses of biological functions and changes in these functions as a result of environmental factors.

There has been significant research on the use of gene expression data to identify people with diseases (disease biomarkers), to monitor exposure to chemicals (exposure biomarkers), and to predict effects from exposure to chemical agents (effects biomarkers). Much of the recent work on effects biomarkers has focused on the classification of genes into ontology groups that can then be used to predict a biological effect (27–30). These efforts can be broken down into two different approaches. In the first approach, the genes from a specific ontology group form a set. If many of the genes in this set have altered gene expression following a chemical exposure, then the effect is significant for that ontology group. Gene set enrichment analysis (GSEA) (31) or some other appropriate approach can be used to determine the significance of a specific effect from gene expression data.

The enrichment analysis approach can be used on gene sets that consist solely of lists of genes. One approach widely used to define gene sets is to base them upon the proteins in signaling or metabolic pathways already described by many years of research. There are numerous collections of pathways that could be used, such as the Kyoto Encyclopedia of Genes and Genomes Pathways (KEGG pathways) (32). The second approach toward the linking of genes to effects is to use the structure of the pathways as well as the membership of gene products in a pathway to determine linkage to the pathway. One example of such an approach is the Structurally Enhanced Pathway Enrichment Algorithm (SEPEA) (33). Gohlke et al. (34) used the SEPEA algorithm to build a linkage model between genes associated with human diseases and the KEGG pathways. Starting with the Genetic Association Database (GAD) (35), they classified human diseases and conditions into 208 broad diseases and disease categories (e.g., liver cancer, epilepsy, and type II diabetes), which we will refer to as the human diseases. For each human condition, they extracted from the GAD all of the genetic polymorphisms associated with that condition and used SEPEA to determine which pathways are most likely to be associated with the disease. This created a linkage mapping between human diseases and the KEGG pathways. To demonstrate the utility of this linkage mapping, Gohlke et al. then used the SEPEA algorithm to link data on changes in gene expression due to chemical exposure from the Comparative Toxicogenomics Database (CTD) (36) with the KEGG pathways. By combining the chemical/pathway and pathway/disease linkages, they were able to predict the known linkages between chemicals/pharmaceuticals/nutrients, and human diseases.

This paper uses a similar approach to identify plausible linkages between exposure to ELF and RF EMF and human diseases. Several authors have looked at changes in gene expression in cellular systems following exposure to EMF using microarrays. We will use these data to find linkages between alterations in gene expression and the KEGG pathways. We will then update the analysis done by Gohlke et al. linking KEGG pathways to human diseases, although using broader disease categories. Given the linkages between EMF and pathways and pathways and human diseases, we will predict plausible linkages between EMF and human diseases. These linkages form hypotheses that can be pursued in other research efforts to study the potential health effects of EMF.

## Materials and Methods

### Gene Expression Data

Microarray data on changes in gene expression in human cells were obtained in three separate ways. In late 2011, we searched the Gene Expression Omnibus using the following search terms: “electromagnetic,” “magnetic,” “electric,” “RF,” “radio,” and “ELF.” This located data from two studies (37, 38). We also did an extensive search of the literature using PubMed, Web of Science, and the EMF Portal^1^ with the same search terms and identified 287 published manuscripts matching these terms. After a review of all of these manuscripts, 22 studies using microarrays for the analysis of EMF effects were identified. Authors were contacted and asked to provide us with data, resulting in data being provided for two studies (39, 40). Of the remaining papers, eight provided complete information on the genes that were significantly altered making them useful for this analysis (41–48). Finally, we were provided the original data created under the EU REFLEX initiative (49, 50). All of the microarray experiments are described below. The descriptions include indications of what measure of gene expression was used as input to the pathway analysis algorithm. Characteristics of the datasets are summarized in Table 1. The datasets used for each analysis are referred to by letter codes that are given at the start of each paragraph below.

**Table 1:** **Studies of gene expression in human cells following exposure to EMF**.

Code	Reference	Field type	Field characteristics	Human cell type	Chip	Samples
A	(39)	RF	900 MHz GSM	SK-N-SH, 0.2 W/kg pulsed	Affymetrix human	2
B	(38)	RF	1800 MHz, 2 W/kg GSM	MCF-7	Affymetrix HU133A	4
C	(38)	RF	1800 MHz, 3.5 W/kg GSM	MCF-7	Affymetrix HU133A	4
D	REFLEX-1 (50)	RF	1800 MHz, 2 W/kg GSM	NB69	Human RZPD-2	4
E	REFLEX-2 (50)	RF	900 MHz, 1.8–2.5 W/kg GSM	EA.hy926 endothelial	Human RZPD-2	4
F	REFLEX-3 (50)	RF	1800 MHz, 1.8–2.5 W/kg GSM	EA.hy926 endothelial	Human RZPD-2	4
G	REFLEX-4 (50)	RF	1800 MHz, 1.8–2.5 W/kg GSM	EA.hy926 endothelial	Human RZPD-2	4
H	REFLEX-5 (50)	RF	1800 MHz, 1.4 W/kg GSM	Quiescent T lymphocytes	Human RZPD-2	4
I	REFLEX-6 (50)	RF	900 MHz, 2 W/kg GSM	U937	Human RZPD-2	4
J	REFLEX-7 (50)	RF	900 MHz, 2 W/kg GSM	CHME5	Human RZPD-2	4
K	REFLEX-8 (50)	RF	1800 MHz, 1 W/kg DTX mode	HL60 leukemia	Human RZPD-2	4
L	REFLEX-9 (50)	RF	1800 MHz, 1.3 W/kg DTX mode	HL60 leukemia	Human RZPD-2	4
M	REFLEX-10 (50)	RF	1800 MHz, 1.3 W/kg DTX mode	HL60 leukemia	Human RZPD-2	4
N	(52)	LF	2080 Hz duty cycle of 90%	Epidermal keratinocytes	Affymetrix HU133A	2
O	(40)	ELF	50 Hz, 1 mT 45 min	Umbilical cord blood monocytes	Human RZPD-2	2
P	REFLEX-11 (50)	ELF	50 Hz, 1 mT 24 h	ES-1	Human RZPD-2	4
Q	REFLEX-12 (50)	ELF	50 Hz, 1 mT 15 h	ES-1	Human RZPD-2	4
R	REFLEX-13 (50)	ELF	50 Hz, 2 mT 16 h	SY5Y	Human RZPD-2	4
S	Pooled (five studies)	RF	Multiple	Multiple	Multiple	5
T	Pooled (three studies)	ELF	50 Hz, multiple	Multiple	Multiple	3

A: Human SK-N-SH neuroblastoma cells were exposed to 900 MHz GSM signals with a specific absorption rate (SAR) of 0.2 W/kg for 2 h (39). Duplicates were pooled for analysis using Affymetrix Human Focus Gene Arrays. Affymetrix IDs were converted to Entrez with an online Gene ID Conversion Tool.^2^ Gene expression data were reported as *p*-values for the significance of change in gene expression between controls and exposed cells; (1 − *p*) was used as the measure of gene expression.

B, C: Human MCF-7 breast cancer cells were exposed to 1800 Hz RF EMF (38). There were two exposure conditions with matched sham controls: 2 W/kg SAR (experiment C) and 3.5 W/kg SAR (experiment D). Exposures were for 24 h. Each group had two biological replicates. Gene expression was measured using Affymetrix human GeneChip HG-U133A. Data were identified by Affymetrix Probe ID, which was converted to Entrez using the same procedures as in A. Log_2_ ratios of control/exposed expression values were used as the measure of gene expression.

D: Human neuroblastoma NB69 cells were exposed to 1800 MHz RF EMF for 24 h (5 min on, 5 min off) at an SAR of 2 W/kg (49, 50). Exposed and control cells were analyzed by Human RZPD-2 microarray. Genes were identified with GenBank IDs and converted to Entrez using IDConverter (51). The mean of the log_2_ of the gene expression ratio for four samples (two samples from two hybridizations) was used as the measure of gene expression.

E–G: Human endothelial EA.hy926 cells were exposed to 900 MHz (experiment F) or 1800 MHz (experiments G and H) RF EMF for 1 h at an SAR of 1.8–2.5 W/kg (49, 50). Exposed and control cells were analyzed by Human RZPD-2 microarray. Genes were identified and analyzed as in D.

H: Human quiescent T lymphocyte cells were exposed to GSM-modulated RF EMF at 1800 MHz, 10 min on and 20 min off, for 44 h at an SAR of 1.4 W/kg (49, 50). Triplicates of exposed and control cells were pooled for analysis by Human RZPD-2 microarray. Genes were identified and analyzed as in D.

I: Human U937 monocytic lymphoma (lymphoblastoma) cells were exposed to 900 MHz RF EMF for 1 h at an SAR of 2 W/kg (49, 50). Exposed and control cells were analyzed by Human RZPD-2 microarray. Genes were identified and analyzed as in D.

J: Human CHME5 microglial cells were exposed to 900 MHz RF EMF for 1 h at an SAR of 2 W/kg (49, 50). Exposed and control cells were analyzed by Human RZPD-2 microarray. Genes were identified and analyzed as in D.

K–M: Human HL60 leukemia cells were exposed to 1800 MHz RF EMF using GSM DTX modulation for 24 h (5 min on, 5 min off) at an SAR of 1 W/kg (experiment L) or for 24 h (continuous) at an SAR of 1.3 W/kg (experiments M and N) (49, 50). Exposed and control cells were analyzed by Human RZPD-2 microarray. Genes were identified and analyzed as in D.

N: Human keratinocytes in cell culture dishes were wounded and exposed to an electric field with a strength of 2.5 mV/cm and a frequency of 2080 Hz for 1 h (52). Triplicate controls and triplicate exposed cells were combined for analysis using the Affymetrix Human Genome HU133A 2.0 GeneChip array. Data were identified by Affymetrix Probe ID, which was converted to Entrez using the same procedures as in A. Log_2_ ratios of control/exposed expression values were used as the measure of gene expression.

O: Monocytes from human umbilical cord blood were exposed to 50 Hz, 1.0 mT ELF EMF for 45 min (40). The available data included exposed/control expression ratios for 998 genes with at least a twofold change in expression (up or down). Genes are identified by gene names, which were converted to Entrez using the same method as in A. Log_2_ of the ratio was used as the measure of gene expression.

P, Q: Human diploid fibroblast cells (ES-1) were exposed to 50 Hz ELF EMF at 1 mT for 5 min on/10 min off for 15 h (P) or 15 h (Q) (49, 50). Exposed and control cells were analyzed by Human RZPD-2 microarray. Genes were identified and analyzed as in D.

R: Human neuroblastoma cells (SH-SY5Y) were exposed to 50 Hz ELF EMF at 2 mT for 5 min on/5 min off for 15 h (49, 50). Exposed and control cells were analyzed by Human RZPD-2 microarray. Genes were identified and analyzed as in D.

S: This pooled dataset combines all significant genes from five RF EMF experiments (41, 43, 44, 46, 47) with complete reporting of all significant alterations in gene expression. Jurkat human T lymphoma cells were exposed to 1763 MHz RF EMF radiation at 2 or 10 W/kg for 30 min (44). The Applied Biosystems 1700 full genome expression human microarray was used to evaluate changes in gene expression following exposure. Human glioblastoma cells (U87MG) were exposed for 4 h to 1.9 GHz pulse-modulated RF EMF at 0.1, 1, and 10 W/kg (47). Changes in gene expression were calculated using the Agilent Human 1A (v1) oligonucleotide 22 K microarray. Human skin fibroblasts (Detroit 550) were exposed to GSM RF EMF at 902.4 MHz for 1 h at an intensity of 0.6 W/kg (46). Gene expression was assessed using the Atlas Human Array Trial kit. A human mast cell line, HMC-1, was exposed to 7 W/kg of 864.3 MHz RF EMF for three exposures each of 20-min duration daily for 7 days (43). Gene expression was assessed using the Atlas Human cDNA Array. Human cell lines, A172 (glioblastoma), H4 (neuroglioma), and IMR-90 (fibroblasts from normal fetal lung), were exposed to 2.1425 GHz continuous wave (CW) and wideband code division multiple access (W-CDMA) RF EMF fields at three field levels: 80, 250, or 800 mW/kg (41). Gene expression changes were determined using the Affymetrix Human genome HG-U133A and B chips. The measure of gene expression used in the pathway analysis was an indicator variable, equal to 1 for genes with significant changes in expression and equal to 0 otherwise.

T: This pooled dataset combines all significant genes from three ELF EMF experiments (42, 45, 48) with complete reporting of all significant alterations in gene expression. Primary human mesenchymal stem cells and a human chondrocyte cell line (C28I2) were exposed to pulsed 50 Hz EMF 8 min per day for up to 3 days with a mean field strength of 35 mT (48). Microarray analyses were performed using the Affymetrix Gene Chip HG-U133A. Peripheral human lymphocytes were exposed to 50 Hz pulsed BEMER-type EMF five times at 12-h intervals for 8 min with a mean field strength of 35 mT (45). Microarray analyses were performed using a custom oligonucleotide array. Cells from a human breast cancer cell line (MCF-7) were exposed to 50 Hz EMF for 24 to 96 h at 1.2 mT (42). Analysis of RNA was performed using custom microarray nylon membranes. The measure of gene expression used in the pathway analysis was an indicator variable, equal to 1 for genes with significant changes in expression and equal to 0 otherwise.

### SEPEA Pathway Analysis

Because the effects of external influences on the body are mediated through changes in cellular function that are themselves controlled by various signaling pathways, it is helpful to analyze data on genetic transcription effects using methods taking those pathways into account. One such method is the SEPEA algorithm (33), which evaluates the degree to which a known genetic pathway is significantly affected by changes in genes or their products. When we describe an exposure as linked to a specific disease, it reflects the significance of this test. Pathway data for humans were downloaded from the KEGG database (32). For purposes of the SEPEA analysis, only 161 of the 206 human pathways were used (pathways with 3 or fewer genes, pathways corresponding to diseases or health conditions, and catchall pathways were excluded).

### Disease–Gene Association

The GAD (35) was downloaded on August 16, 2010. Genes indicated as being associated with diseases were used in the analysis. A total of 16,621 gene–disease associations were found, with 3562 unique gene names. IDConverter^3^ was used to match gene names to Entrez IDs, and 3172 of the gene names were matched. The disease classes listed in the GAD were used for the analysis. Of the 19 disease categories, categories “Mitochondrial,” “Normal variation,” “Other,” “Unknown,” and “Pharmacogenomic” were not used in the analysis due to few linked genes. The 14 disease classes used in the analysis and the numbers of matched genes associated with them are shown in Table 2.

**Table 2:** **Number of pathways linked to disease class in the Genetic Association Database using the SEPEA algorithm**.

Disease class	Number of genes	Number of pathways
Aging	67	4
Cancer	549	21
Cardiovascular	649	17
Chemical dependency	300	11
Developmental	346	4
Hematological	209	6
Immune	708	16
Infection	201	12
Metabolic	770	22
Neurological	497	10
Psychological	460	11
Renal	126	5
Reproduction	180	12
Vision	80	5

Disease–gene associations were used as input to the SEPEA algorithm. The association was considered to be significant if *p* < 0.01. This is similar to the earlier analysis (34) with broader disease categories and the most recent information contained in the GAD. The numbers of pathways associated with each disease are presented in Table 2.

### EMF-Pathway Linkage

The significance of associations between experimental results and KEGG pathways were derived by using the SEPEA algorithm. For this analysis, pathways were assumed to be associated with EMF exposure if *p* < 0.05. All individual experiments (A–T) were analyzed against the 162 KEGG pathways. The version of the SEPEA algorithm used in this analysis was SEPEA NT3, which requires only one data point, a measure of strength of expression, for each gene. Where full data on gene expression levels were available, we used log_2_ of the exposed/control expression ratio. When full data were not available, we used different measures of expression strength, as noted in the data descriptions above.

Several additional pooled analyses were done to evaluate linkages to pathways and diseases across a broad array of exposures and cell lines. We pooled all complete RF EMF microarray datasets (A–M) into one dataset for analysis, all complete ELF EMF microarray datasets (O–R), all RF EMF experiments (A–M, S), all ELF EMF experiments (O–R, T), and all experiments (A–T).

### EMF-Disease Linkage

Significant association between the gene expression changes from a single dataset using EMF exposure and each disease was calculated using the hypergeometric function. Under the null hypothesis that the pathways significant in the dataset are unrelated to those significant in the disease, the *p*-value for significant relationship between the dataset and disease was calculated. If there are *N* pathways for the disease and *K* for the dataset, with *M* total pathways, and there were *X* pathways in common, then the probability of at least *X* in common was calculated [1 − H(*X* − 1,*M*,*K*,*N*)], where H(*X* − 1,*M*,*K*,*N*) is the hypergeometric CDF at *X* − 1 for drawing *K* items in *N* drawings without replacement from a set of *M* objects.

## Results

This research used microarray data from the literature to predict linkages between RF EMF or ELF EMF with human disease categories. The linkage was done in three steps: (1) linkage of human disease categories to KEGG pathways using genetic polymorphisms, (2) linkage of EMF exposure with KEGG pathways using gene array data, and (3) comparison of the disease-linked pathways with EMF-linked pathways to predict significant linkages between EMF and human disease.

### Human Disease to KEGG Pathway Linkage Analysis

Table 2 provides the number of significant pathways found for each human disease. Pathways ranged from as few as 4 for aging and developmental disorders to as high as 21 for cancer and 22 for metabolic disorders. Aging, developmental (four pathways), renal, vision (five pathways), hematological (six pathways) disorders, immune disorders, and infection had few significant pathways and will not be discussed further. The full listing of pathways associated with each human disease is given in Spreadsheet S1 in Supplementary Material.

Cancer was linked with 21 pathways, predominantly relating to metabolism (7 pathways), hormone control (2 pathways), DNA repair (3 pathways), and cellular replication (3). Other pathways significantly linked to cancer and known to be important to carcinogenesis included JAK–STAT signaling and adipocytokine signaling. Thus, of the 21 pathways identified, 17 have long-standing linkages to cancer as a disease process.

Cardiovascular disease was linked to 17 pathways, 5 associated with metabolism of xenobiotics and hormones, 5 pathways associated with inflammatory response, and 4 linked pathways relating to cellular homeostasis and control (calcium signaling, gap junction management, neuroactive ligand–receptor interactions, and aldosterone-regulated sodium reabsorption).

Chemical dependency was significantly linked to 11 pathways; 6 metabolic pathways, 4 linked pathways relating to cellular homeostasis and control (glucogenesis, calcium signaling, neuroactive ligand–receptor interactions, and long-term potentiation), and NOD-like receptor signaling involved in immune response.

There were 10 pathways linked to neurological disorders. Psychological disorders had 11 linked pathways, 4 of which were shared with neurological disorders, all linked to basic cellular control and homeostasis (tyrosine metabolism, calcium signaling, neuroactive ligand–receptor interactions, and the renin angiotensin system). Both disorders had additional metabolic pathway linkage, with psychological disorders additionally linked to metabolism of linoleic acid and arachidonic acid (three essential fatty acids), while neurological disorders were linked to cytochrome P450 metabolism. Neurological disorders were linked to PPAR and Notch signaling pathways, both important in neurological development. Psychological disorders were also linked to circadian rhythm control and long-term depression, a signaling network thought to be a molecular and cellular basis for cerebellar learning with multiple signal-transduction pathways involved in this process.

Metabolic disorders were associated with 22 pathways, 13 of which deal with metabolism of drugs, xenobiotics, lipid, fatty acids, and steroids. The remaining pathways included the ATP-based transporters, basic cellular control and homeostasis pathways, PPAR, insulin signaling, and adipocytokine signaling, many of which have already been associated with metabolic disorders.

The 12 pathways associated with reproductive disorders covered a variety of systems, some of which were related to basic cellular functions and others dealing with a variety of pathways not usually associated with reproductions. Because of the diversity of this response, it will not be further discussed.

In total, 60 of the 162 pathways were associated with at least 1 human disease or disorder. Many of these were linked to five or more diseases and dealt with basic cellular functions like JAK–STAT signaling, cytokine–cytokine receptor interaction, and neuroactive ligand–receptor interaction. Many of the other multi-disease pathways involve metabolic functions. However, there were several specific to certain diseases; DNA repair pathways and cancer, circadian rhythm and psychological diseases, and insulin signaling and several metabolic pathways specific to metabolic disorders. All of the evaluations for all pathways and diseases are presented in the Spreadsheet S1 in Supplementary Material.

### EMF Exposure to KEGG Pathway Linkage Analysis

In this study, 13 RF EMF (A–M) microarray, 1 LF EMF (N) microarray, and 4 ELF EMF (O–R) microarray datasets with human cells for which we had the original data were analyzed. In addition, two datasets were constructed by pooling five RF EMF (dataset S) and three ELF EMF (dataset T) microarray studies from the literature in multiple cell lines with multiple field strengths and exposure durations. These literature studies identified all significantly altered genes.

As a general rule, the individual datasets were linked to biologically diverse pathways. The total number of linked pathways was also quite diverse ranging from 3 for dataset P to 26 for dataset N. The separate analyses of the individual RF EMF studies showed 31 pathways being significant at *p* < 0.05 in more than one study. When all of the RF EMF studies are combined in one analysis, there were 25 significant pathways linked to the exposure.

The LF study (N) was significant for every disease/disorder category. The ELF EMF studies did not demonstrate a response as robust as the LF and RF EMF studies. The four individual studies, for which we had complete data (O–R), showed 3 to 14 linkages each. Only two pathways were significantly linked in more than one study, both being linked in two studies. These two pathways, inositol phosphate metabolism and FC gamma R-mediated phagocytosis are not related. The analysis of the LF and ELF EMF data combined (experiments N–R, T) resulted in 15 significantly linked pathways. No obvious pattern emerged from these linked pathways.

All of the linkages between the experiments and the KEGG pathways are provided in Spreadsheet S2 in Supplementary Material.

### EMF Exposure Linkage to Human Disease through Associated KEGG Pathways

Ten (A, E–F, H–M) of the 13 RF EMF studies demonstrated no significant linkages to disease based on common pathways. Dataset B demonstrated the strongest pathway linkages to disease with significant linkages to three disease categories; cardiovascular disease, psychological disorders, and reproduction. The identical study using a higher power exposure (C) was significantly linked to chemical dependency, but to none of the same linkages seen for B. Dataset G was significantly linked to reproductive disorders resulting in a total of 2 of the 13 datasets linked to this disease category.

Cardiovascular disease was linked to the significant pathways from only dataset B (seven pathways) and did not show any additional linkage, even in the combined datasets. Similarly, psychological and reproductive disorders were significantly linked to study B (five and four pathways, respectively) but did not demonstrate significant linkage in the combined analyses.

Reproductive disorders show significant linkages to two pathways for dataset G with only one pathway occuring in dataset B (long-term depression). However, when the datasets were combined (A–M, S), the significance could not be maintained because the combined dataset only linked to one pathway, long-term depression.

Cardiovascular disease was significantly linked in dataset B to five linked pathways (linoleic acid metabolism, calcium signaling pathway, neuroactive ligand–receptor interaction, intestinal immune network for IgA production, and salivary secretion). In contrast, study C was not significantly linked with cardiovascular disease because of only two pathways (calcium signaling pathway and neuroactive ligand–receptor interaction), both also linked in dataset B. The combined analysis using all of the RF EMF microarray data had no linkage to cardiovascular disease.

Chemical dependency was significantly linked to only one dataset (C) with three pathways (histidine metabolism, calcium signaling pathway, and neuroactive ligand–receptor interaction). No linkage was seen in the combined analysis.

When the RF data are combined (A–M and A–M, S), there are no significant linkages to diseases or disorders.

Study N (LF) was linked to all seven diseases and disorders in Table 3. It was the only study to identify a significant linkage with cancer (9 pathways; steroid hormone biosynthesis, linoleic acid metabolism, retinol metabolism, metabolism of xenobiotics by cytochrome P450, drug metabolism – cytochrome P450, drug metabolism – other enzymes, JAK–STAT signaling pathway, hematopoietic cell lineage, and long-term potentiation), metabolic disorders (10 pathways; steroid hormone biosynthesis, starch and sucrose metabolism, linoleic acid metabolism, retinol metabolism, metabolism of xenobiotics by cytochrome P450, drug metabolism – cytochrome P450, drug metabolism – other enzymes, ABC transporters, neuroactive ligand–receptor interaction, and long-term potentiation), and neurological disorders (6 pathways; tyrosine metabolism, metabolism of xenobiotics by cytochrome P450, drug metabolism – cytochrome P450, calcium signaling pathway, neuroactive ligand–receptor interaction, and hematopoietic cell lineage).

**Table 3:** ***p*-Values linking single and combined experiments to various disease and disorder categories through SEPEA**.

Diseases studies	Field	Cancer	Cardiovascular	Chem.-dependency	Metabolic	Neurological	Psych	Reproduction	# Linked pathways
A	RF	0.5921	–	0.3765	–	–	–	–	6
B		0.5561	0.0055^a^	0.2503	0.2535	0.1872	0.0005^b^	0.0124^c^	13
C		–	0.2849	0.0284^d^	0.7803	0.1196	0.1413	0.5800	10
D		–	–	0.5856	0.8123	–	–	–	11
E		0.2447	–	–	0.2447	–	–	0.4518	8
F		0.8123	0.7191	–	–	–	–	–	12
G		0.4477	–	–	0.4477	–	0.2486	0.0331^e^	5
H		0.5921	–	–	–	–	0.3505	–	6
I		–	–	–	–	–	–	–	4
J		0.6500	–	–	–	–	–	–	7
K		0.5095	0.3689	–	0.8398	–	0.5856	–	12
L		–	–	–	–	0.1639	0.5856	0.6494	12
M		–	–	–	0.5921	–	–	–	6
N	LF	0.0024^f^	0.0082^g^	0.0006^h^	0.0004^i^	0.0014^j^	0.0175^k^	0.0078^l^	26
0	ELF	0.7803	0.2849	–	0.7803	–	0.5176	–	10
P		–	–	–	–	–	–	–	3
Q		0.5997	0.4499	0.6779	0.1035	–	–	0.7081	14
R		–	–	–	–	–	–	–	3
S	RF	0.4085	0.2849	–	–	–	0.5176	0.5800	10
T	ELF	0.0082^m^	0.0557	0.0282^n^	0.0082^o^	0.0196^p^	0.2486	–	4
A–M	RF	0.9288	–	–	–	0.6835	0.7191	0.7794	25
A–M, S		0.9288	–	–	–	0.6835	0.7191	0.7794	25
N–R	ELF	0.0760	0.1022	0.0005^q^	0.0202^r^	0.0152^s^	0.1093	0.0415^t^	14
N–R, T		0.0935	0.1206	0.0001^u^	0.0059^v^	0.0187^w^	0.3813	0.1850	15
A–T	All	0.0935	0.0329^x^	0.0375^y^	0.0935	0.0187^z^	0.1248	–	19

*^a^Linoleic acid metabolism; calcium signaling pathway; neuroactive ligand–receptor interaction; intestinal immune network for IgA production; salivary secretion*.

*^b^Tyrosine metabolism; linoleic acid metabolism; calcium signaling pathway; neuroactive ligand–receptor interaction; long-term depression*.

*^c^Steroid hormone biosynthesis; metabolism of xenobiotics by cytochrome P450; neuroactive ligand–receptor interaction; JAK–STAT signaling pathway; hematopoietic cell lineage; long-term depression*.

*^d^Histidine metabolism; calcium signaling pathway; neuroactive ligand–receptor interaction*.

*^e^One carbon pool by folate; long-term depression*.

*^f^Steroid hormone biosynthesis; linoleic acid metabolism; retinol metabolism; metabolism of xenobiotics by cytochrome P450; drug metabolism – cytochrome P450; drug metabolism – other enzymes; JAK–STAT signaling pathway; hematopoietic cell lineage; long-term potentiation*.

*^g^Linoleic acid metabolism; retinol metabolism; metabolism of xenobiotics by cytochrome P450; calcium signaling pathway; neuroactive ligand–receptor interaction; hematopoietic cell lineage; salivary secretion*.

*^h^Tyrosine metabolism; retinol metabolism; metabolism of xenobiotics by cytochrome P450; drug metabolism – cytochrome P450; calcium signaling pathway; neuroactive ligand–receptor interaction; long-term potentiation*.

*^i^Steroid hormone biosynthesis; starch and sucrose metabolism; linoleic acid metabolism; retinol metabolism; metabolism of xenobiotics by cytochrome P450; drug metabolism – cytochrome P450; drug metabolism – other enzymes; ABC transporters; neuroactive ligand–receptor interaction; long-term potentiation*.

*^j^Tyrosine metabolism; metabolism of xenobiotics by cytochrome P450; drug metabolism – cytochrome P450; calcium signaling pathway; neuroactive ligand–receptor interaction; hematopoietic cell lineage*.

*^k^Drug metabolism – cytochrome P450; Notch signaling pathway*.

*^l^Steroid hormone biosynthesis; metabolism of xenobiotics by cytochrome P450; neuroactive ligand–receptor interaction; JAK–STAT signaling pathway; hematopoietic cell lineage; long-term depression*.

*^m^Linoleic acid metabolism; retinol metabolism; drug metabolism – cytochrome P450*.

*^n^Retinol metabolism; drug metabolism – cytochrome P450*.

*^o^Linoleic acid metabolism; retinol metabolism; drug metabolism – cytochrome P450*.

*^p^Drug metabolism – cytochrome P450; Notch signaling pathway*.

*^q^Glycolysis/gluconeogenesis; retinol metabolism; metabolism of xenobiotics by cytochrome P450; drug metabolism – cytochrome P450; calcium signaling pathway; neuroactive ligand–receptor interaction*.

*^r^Steroid hormone biosynthesis; retinol metabolism; metabolism of xenobiotics by cytochrome P450; drug metabolism – cytochrome P450; drug metabolism – other enzymes; neuroactive ligand–receptor interaction*.

*^s^Metabolism of xenobiotics by cytochrome P450; drug metabolism – cytochrome P450; calcium signaling pathway; neuroactive ligand–receptor interaction*.

*^t^Steroid hormone biosynthesis; metabolism of xenobiotics by cytochrome P450; neuroactive ligand–receptor interaction; long-term depression*.

*^u^Glycolysis/gluconeogenesis; retinol metabolism; metabolism of xenobiotics by cytochrome P450; drug metabolism – cytochrome P450; calcium signaling pathway; neuroactive ligand–receptor interaction; NOD-like receptor signaling pathway*.

*^v^Steroid hormone biosynthesis; starch and sucrose metabolism; retinol metabolism; metabolism of xenobiotics by cytochrome P450; drug metabolism – cytochrome P450; drug metabolism – other enzymes; neuroactive ligand–receptor interaction*.

*^w^Metabolism of xenobiotics by cytochrome P450; drug metabolism – cytochrome P450; calcium signaling pathway; neuroactive ligand–receptor interaction*.

*^x^Linoleic acid metabolism; retinol metabolism; metabolism of xenobiotics by cytochrome P450; neuroactive ligand–receptor interaction; hematopoietic cell lineage*.

*^y^Retinol metabolism; metabolism of xenobiotics by cytochrome P450; drug metabolism – cytochrome P450; neuroactive ligand–receptor interaction*.

*^z^Metabolism of xenobiotics by cytochrome P450; drug metabolism – cytochrome P450; neuroactive ligand–receptor interaction; hematopoietic cell lineage*.

Dataset N linked with seven pathways for cardiovascular disease, three of which matched what was seen for dataset B (linoleic acid metabolism, neuroactive ligand–receptor interaction, and salivary secretion). Although dataset B linked with seven pathways for chemical dependency, only two matched dataset C (calcium signaling pathway and neuroactive ligand–receptor interaction). Dataset N was linked to five pathways for psychological disorders, four of which matched dataset B (linoleic acid metabolism, calcium signaling pathway, neuroactive ligand–receptor interaction, and long-term depression). Finally, dataset N linked to six pathways for reproductive disorders, matching both datasets B and G for long-term depression pathway and matching dataset B for neuroactive ligand–receptor interaction.

None of the ELF EMF datasets (O–R) were significantly linked to any disease. Surprisingly, the combined ELF EMF literature dataset (T) was linked to cancer, chemical dependency, metabolic disorders, and neurological disorders, predominantly through linoleic acid metabolism, retinol metabolism, and drug metabolism by cytochrome P450. The combined LF/ELF EMF datasets (N–R, T) were significantly linked to chemical dependency, metabolic disorders, and neurological disorders, predominantly by datasets N and T.

Combining all of the data from RF, LF, and ELF resulted in significant linkages to cardiovascular disease, chemical dependency, metabolic disorders, and neurological disorders.

## Discussion

### Pathways and Processes Impacted by RF EMF Exposure

The strongest linkage between RF EMF and the KEGG pathways relates to cellular structure and cytoskeleton maintenance. Extra cellular matrix (ECM)–receptor interaction relates to the complex mixture of structural and functional macromolecules that help to regulate cellular structure and plays an important role in tissue and organ morphogenesis. Although this pathway was unrelated to any disease based upon our linkage analysis, it was linked to six of the RF EMF datasets. This pathway links to other KEGG pathways, including the focal adhesion pathway (four datasets linked). The focal adhesion pathway represents the signaling associated with cell membrane to extracellular matrix contact points through transmembrane receptors of the integrin family. Focal adhesion was significantly linked in four of the same datasets as ECM–receptor interaction. The findings cross multiple cell lines, although there is insufficient data to isolate any cell line as responsive or not. Both pathways were significant in both pooled datasets (A–M and A–M, S).

Previous studies found evidence for linkage between cytoskeleton development and RF EMF. Changes in two vimentins, intermediate filament proteins that make up part of the cytoskeleton, were observed in a proteomics analysis of endothelial cells following RF EMF exposure (53). Gene expression changes in microtubule-associated proteins 2, 1b, and tau, genes that control the development of microtubules (54), were also observed. Exposure of embryonic neural stem cells at 4 W/kg 1800 MHz inhibited the neurite outgrowth and reduced the mRNA and protein expression of the proneural genes Ngn1 and NeuroD, whereas the expression of Hes1, an inhibitor or neurite outgrowth was decreased (55). Finally, a fourfold increase in beta15 thymosin in rat primary cortical neurons was seen after RF EMF exposure and was associated with a change in neurite branching (56).

Changes in the ECM–receptor interaction and focal adhesion are linked to a variety of higher level cellular functions, such as apoptosis and cell cycle control. Thus, if one were seeing changes in these pathways, it would be likely that changes would be seen in related pathways that are part of the apoptotic process and/or cell cycle control. Of the eight pathways with significant linkages in three or more datasets, three are related to apoptosis. In addition to ECM–receptor interaction and focal adhesion, TGF-β signaling (5), apoptosis (3), and p53 signaling (3) are all significantly linked in multiple studies. Apoptosis, a basic cellular function, is linked to cancer and several other diseases. These three pathways are all linked to cell cycle control, suggesting that RF EMF can interfere with the routine cellular functions associated with cell cycle control; cytoskeleton and extracellular matrix management, apoptosis, and cellular replication. There is evidence from single endpoint studies reporting changes in gene and/or protein expression after RF EMF exposure related to apoptosis and/or cell cycle control (10, 15, 16, 19, 20, 23, 24, 57, 58).

These findings are expected from the literature. MAPK signaling (two studies) plays an important role in all of these processes and appears extensively in these pathways. The ERK 1 and 2 are MAPKs that function in cellular proliferation, differentiation, and survival, and their inappropriate activation is a common occurrence in human cancers. Several authors (15, 16, 19, 24, 25, 46, 59) have shown changes in ERK (either transcription or activity), following exposure to RF EMF fields. The c-Jun N-terminal kinases (JNKs) are also MAPKs regulated by specific MAPK kinases (MKKs) and MKK kinases (MKKKs) that phosphorylate and regulate the activity of transcription factors and regulatory proteins in the cell. JNKs are regulated by growth factors, cytokines, cell adherence, and stress stimuli. These MAPKs also appear in most of the pathways mentioned above and also have an extensive literature showing modification by RF EMF (16, 19, 24, 25). Finally, the p38 MAPKs are involved in cellular replication and differentiation and, along with the JNKs, are responsive to stress stimuli, such as mitogens, ultraviolet radiation, and heat shock. These have been extensively studied in the RF and ELF EMF literature with mixed results where some researchers have seen changes in p38 MAPKs following RF EMF exposure (19), while others have not (5, 15, 16, 24, 25), even though most of the studies demonstrated changes in heat-shock proteins.

Additional support for these findings comes from other studies in the literature, including studies evaluating changes in p53 (19, 20) and changes in c-fos and other immediate early genes (58, 60–63) that had varied results (58, 64).

The other strong linkage identified in our transcriptomics analyses of human cells exposed to RF EMF relates to metabolic pathways linked to some of the datasets. Unlike apoptosis, cell cycle control, and cellular structure, the linkages to metabolic processes generally involved only one or two databases and hence represent a weaker association. One pathway related to carbohydrate metabolism (galactose metabolism) and two pathways related to lipid metabolism (fatty acid elongation and primary bile acid biosynthesis) were each linked to two datasets. Many of the original publications using gene ontology analyses noted significant linkages to metabolism (26, 65). Lipid metabolism may involve STAT3 activation. Activators of the JAK–STAT pathway include cytokines and growth factors. JAKs mediate the recruitment of MAPK leading to cell cycle changes, apoptosis, differentiation, or lipid metabolism. The release of pro-inflammatory cytokines was demonstrated in microglia and astrocytes after exposure to RF EMF. In microglia, STAT3 activation was in microglia, but not in astrocytes (55, 66, 67). It is worth noting that changes in lipid metabolism can result in cellular stress (68), relating metabolic changes to the earlier changes in cellular response to stress.

### Diseases and RF EMF Exposure

The lack of a strong linkage of RF EMF to cancer and metabolism disorders is surprising. The pathways and processes linked to the RF EMF datasets would suggest a strong linkage to both of these disease classes.

Cancer has been the major focus of studies in humans concerning RF EMF exposure. A scientific panel convened by the International Agency for Research on Cancer concluded it is a possible human carcinogen (1). In this study, in order for cancer to link with RF EMF exposure, two things must occur: there must be a linkage between a set of pathways and cancer and the same set of pathways need to be linked to RF EMF. While we found RF EMF linkage to historical cancer pathways, these same pathways did not link to cancer in the GAD linkage analysis. Of the five pathways linked to apoptosis and cell cycle control, in three or more datasets, only p53 signaling was linked to cancer in the GAD. It could be true that these pathways, such as TGFβ signaling, are not linked to cancer, but this is unlikely based on historical research. It is more likely that genetic polymorphisms of genes in these pathways have not been adequately studied for cancer linkage and hence do not appear in the GAD. For example, the MAPKs have very few entries in the GAD. Alternatively, these pathways may be so basic that genetic variants in genes in these pathways tend to be dominant lethal. It is also possible that our assumption that polymorphisms can be used to identify pathways important in disease occurrence is incorrect.

As for cancer, there was very little linkage between RF EMF and metabolic disorders despite linkage between RF EMF and metabolic pathways. In the GAD, most of the linkages between metabolism genes and diseases are with cancer. These genes were predominantly identified in occupational cohorts where there are gene–environment interactions (69). More recently, genome-wide association studies are beginning to identify additional polymorphisms in diseased populations. However, these studies have not yet been incorporated into the GAD. Even with this bias, the pathways linked to metabolic disorders in the GAD linkage analysis are reasonable and cover the obvious contributors such as tryptophan metabolism. But the linkage between RF EMF and metabolic pathways is too sporadic to provide a significant linkage with the disease pathways. This might also be due to the variability of cells used and therefore, any EMF cell type-specific effects could have been “averaged out” across the diverse cell types. A larger study using more microarrays and targeted cell lines could strengthen this relationship and identify key metabolic pathways that tie RF EMF to disease.

Radiofrequency electromagnetic fields alterations in metabolism are of growing interest due to a few studies showing changes in brain metabolism following RF EMF exposure (70–72). Note, the latter two studies show effects in opposite directions complicating this picture and demonstrating the need for additional research (73).

A complete loss of pyramidal cells in the CA1 area of the hippocampus in mouse brain after 5-day exposure to RF EMF was reported recently (74). Calcium-binding proteins were observed to be changed, and the authors hypothesized these changes would alter cellular calcium levels and possibly be responsible for the deleterious effect on the hippocampus. Frequency-dependent modifications of calcium spikes were seen in P19-derived neuronal cells (75). They found that both N-type calcium channels and phospholipase enzymes appear to be involved in the calcium spiking. Our analysis points in a similar direction with the calcium signaling pathway (this pathway includes phospholipases) linked to neurological disorders in three of our datasets.

### Pathways and LF/ELF EMF Exposures

The LF EMF exposure study (N) demonstrated numerous linkages to pathways, predominantly metabolic pathways. The individual ELF EMF studies O–R demonstrated sporadic linkages to multiple pathways with only three common linkage seen in exactly two studies including cell cycle pathway (Q and R), inositol phosphate metabolism (O and P), and FC gamma R-mediated phagocytosis (O and P). When all of the LF and ELF datasets are combined (N–R and T), there are three additional pathways that were common to two of the studies; drug metabolism *via* cytochrome P450, linoleic acid metabolism, and retinol metabolism (N and T). There is insufficient consistency in these findings to draw any strong conclusions.

### Diseases and LF/ELF EMF Exposures

The LF EMF exposure study (N) was linked to all of the disease classes listed in Table 3, mostly due to significant linkages to numerous metabolic pathways. None of the individual ELF exposure studies (O–R) were linked to any individual disease classes. When combined with the LF exposure study, significant linkages were found for diseases related to chemical dependency, metabolic dysfunction, neurological disorders, and reproduction. The combined ELF literature studies (T) to cancer, chemical dependency, metabolic dysfunction, and neurological disorders. When all LF and ELF exposure studies are combined (N–R, T), significant linkages remained for chemical dependency, metabolic dysfunction, and neurological disorders. However, the lack of linkage for any of the individual studies (O–R) and the strong influence of the LF exposure study on these results preclude any strong conclusions regarding the overall linkages to any disease from these data.

### Biases

There are two aspects of potential bias to consider in this analysis: (1) cell type bias and (2) dividing cell bias in using cell lines. There are a preponderance of neurological cell lines, such as SK-N-SH, NB69, CHME5, and SH-SY5Y, which may favor detection of changes in neurological pathways and disease outcomes. This could bias the conclusion that RF EMF data suggest further study into neurological disorders. Also, the use of cell lines might favor detecting changes in cell division, extracellular matrix, and activation of many signaling pathways compared to more quiescent, non-dividing, differentiated cells. These changes in dividing cells with an active cell cycle and correspondingly engaged signaling and metabolic pathways may favor changes leading to conclusions of outcome diseases like cancer, any type of metabolic disorder, immunological effects, or damage and repair responses to disease. However, just as likely, active cell cycle and correspondingly engaged signaling and metabolic pathways may hide effects on subtle changes in cell cycle regulation that would be detectable and significant in quiescent cells like neurons.

## Conclusion

Our analysis supports a linkage between RF EMF exposure to human cells and changes in the pathways associated with apoptosis, cellular regulation, and cytoskeleton maintenance. There is weaker support for linkage to metabolic pathways and neurological pathways. Based on these linkages alone, there is reason to believe that RF EMF could play a role in carcinogenesis, metabolic disorders, and neurological development and function. The strength of the evidence linking pathways for disease to the RF EMF-linked pathways is weaker. There is little support for a direct linkage between cancer pathways and RF EMF pathways, probably due to the quality of the GAD database. Similarly, there is very little linkage to metabolic disease.

Our analysis supports a linkage between ELF EMF and cancer, chemical dependency, metabolic dysfunction, and neurological disorders; however, these findings are largely driven by a single study and should be considered weak.

The greatest strength of this analysis is that it is fully objective in its approach. Data were identified by literature review; all data were handled equally depending on the type of data available for the analysis. The results are all tied to objective statistical methods that demonstrate the strength of the linkage between various pathways and EMF exposure and disease. The major weakness of this analysis is the inability of these small EMF microarray datasets to provide the depth and complexity to support a more thorough analysis. Also weakening this analysis are the limitations to the GAD as discussed above. Thus, at best, this analysis generates hypotheses that may be followed up. Changes in gene expression do not always correlate to changes in the proteins, enzymes, and transcription factors that govern cellular signaling and cellular metabolism. In following up with further research, both gene expression studies and studies of protein changes should be considered.

## Author Contributions

All authors contributed to the analysis and interpretation of the data in this manuscript. The original idea and work plan for this research was the work of MM and CP.

## Conflict of Interest Statement

The authors declare that the research was conducted in the absence of any commercial or financial relationships that could be construed as a potential conflict of interest.

## Footnotes

^1^http://www.emf-portal.de

^2^http://david.abcc.ncifcrf.gov/conversion.jsp

^3^https://idconverter.bioinfo.cnio.es
